# Natural selection on reproductive timing varies by education in twentieth-century Estonia

**DOI:** 10.1017/ehs.2025.10019

**Published:** 2025-09-08

**Authors:** Richard Meitern, Peeter Hõrak

**Affiliations:** Department of Zoology, Institute of Ecology and Earth Sciences, University of Tartu, Tartu, Estonia

**Keywords:** age of first birth, age of last birth, educational attainment, interbirth interval, natural selection, sexually antagonistic selection

## Abstract

This register-based study investigates how natural selection acts on educational attainment and reproductive timing among Estonians born between 1925 and 1977. Women with primary education consistently achieved the highest reproductive success throughout the study period, whereas a positive educational gradient in reproduction emerged among men born since the 1950s, resulting in sexually antagonistic selection on educational attainment. Men with tertiary education had higher reproductive success than other men, despite initiating reproduction later. The lowest-educated women exhibited the strongest selection for early reproduction and the earliest start of reproduction throughout the study period. These women and the least-educated men also experienced the strongest selection for delayed reproductive cessation. Nonetheless, parents with primary education (particularly men) were typically the first to stop reproducing. Stabilizing selection for intermediate interbirth intervals also showed the strongest quadratic selection gradients among minimally educated parents of both sexes. At that, men with primary education had the fastest reproductive pace, whereas women in the same group had the slowest. Our study shows that selection on reproductive timing traits was consistently stronger among parents with lower educational attainment, and that variation in reproductive timing across educational strata does not consistently reflect the selective pressures acting on recent generations.

## Graphical abstract

**Figure:**
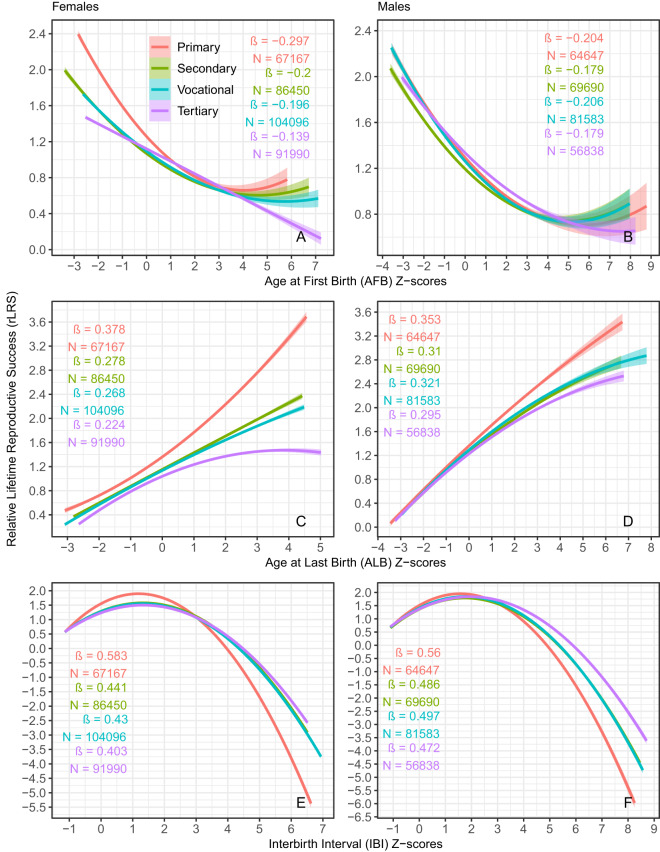

## Social media summary

Educational differences in reproductive timing do not consistently reflect selective pressures in recent generations.

## 1. Introduction

Humans exhibit considerable variation in the timing of their reproductive events, including the ages at which they have their first and last child and the reproductive rates. These traits are influenced by a complex interplay of biological, environmental and sociocultural factors and are subject to natural selection. From an evolutionary perspective, reproductive timing is crucial, as it relates directly to the number and quality of offspring produced, ultimately shaping lifetime reproductive success (Gillespie et al., 2013; Hayward et al., 2015; Scranton et al., 2016).

The intensity and direction of natural selection can vary across populations and subgroups due to variations in ecological, cultural and socioeconomic contexts (G. Andersson et al., 2009; Pettay et al., 2007; Tropf, Barban, et al., 2015). For instance, Pettay et al. (2007) found the strongest selection on earlier age at first reproduction in women of the poorest wealth class, whereas selection favoured older age at reproductive cessation in mothers of the wealthier classes in pre-industrial Finland. A recent UK Biobank study showed most intense natural selection on polygenic scores of anthropometric and behavioural traits among people with lower income and education, younger parenthood, and more lifetime sexual partners. In contrast, among older parents, the direction of natural selection was reversed (Hugh-Jones & Abdellaoui, 2022). Higher levels of education in women are strongly linked to reproductive behaviour, typically leading to delayed childbearing and lower overall fertility (Beaujouan et al., 2023; Berrington et al., 2015; Hellstrand et al., 2024; Kirk et al., 2001; Nisén et al., 2021; Sobotka, 2017; Van Bavel et al., 2018). However, the detailed knowledge of how natural selection relates to reproductive timing across educational strata over extended periods remains limited.

This study examines how natural selection acts on the ages at first and last birth and the birth spacing across different educational levels among Estonian men and women born between 1925 and 1977. Our aims are (1) to describe the secular trends in reproductive success and timing across sexes and educational strata during the 52-year period covering major societal, demographic and educational transformations, and (2) to analyse whether the strength and direction of natural selection on reproductive timing traits varies across sexes and educational strata. Specifically, we will test whether the strength of natural selection is stronger in lower educational levels as shown for polygenic scores of many anthropometric, behavioural and health-related traits in the UK (Hugh-Jones & Abdellaoui, 2022) and US (Hugh-Jones & Edwards, 2024) biobanks.

Unlike most earlier studies (but see Bolund et al., 2013; Bürkli & Postma, 2014; Pettay et al., 2005), we will also examine the effects of natural selection on the reproductive timing of men. Among pre-industrial Finns, phenotypic selection gradients on four traits related to timing and rate of reproduction differed between sexes (Bolund et al., 2013). A previous study on a sample of Estonian children born between 1937 and 1962 revealed strong sexually antagonistic selection on educational attainment (Valge et al., 2022). Building on this, we examined whether similar patterns can be observed over a longer time span using whole-population data, and whether selection on reproductive timing exhibits sex-specific differences.

The birth cohorts in our study experienced significant shifts in external conditions (see Section 2.1) that could have influenced the evolution and/or expression of life-history traits (reviewed by Mawass et al., 2022). These include increasing birth-time economic hardships during World War II and particularly in the post-war period, and decreasing infant mortality (a marker of the epidemiologic situation) after 1948 (Hõrak & Valge, 2015). Between birth cohorts of 1922 and 1959, life expectancy at birth increased from 48.7 to 64.3 years in men and from 54.9 to 71.6 years in women, while from 1965 to 1975 birth cohorts life expectancy of men stagnated around 65 years whereas that of women remained around 73–75 years (Meitern & Hõrak, 2024; Puur et al., 1999). The cohorts under study experienced a significant expansion of secondary, vocational and tertiary education, which gradually surpassed the prevalence of primary/basic education, especially among women ((Klesment, 2013); Supplementary Fig. S1).

We begin by describing general time trends in the variation of completed fertility rates and reproductive timing parameters across birth cohorts at the population level. We then examine natural selection on traits related to the timing and rate of reproduction across sexes and educational strata to determine whether patterns of reproductive timing in different population subgroups reflect the selection pressures acting within those subgroups.

## 2. Methods

### 2.1. Demographic background

The historical background of the study population is briefly summarized by Hõrak and Valge (2015). Of note, total World War II population losses in Estonia were estimated to be among the highest proportions in Europe (Misiunas & Taagepera, 1993). Altogether, out of a population of 1.1 million, about 47,000 were arrested for political reasons, and 35,000 were deported during the period of Stalinist repression (Mertelsmann & Rahi-Tamm, 2009). The terror of the first Soviet year motivated approximately 75,000–80,000 Estonians to flee to the West between 1941 and 1944 under threat of the return of the Soviet regime (Tammaru et al., 2010).

The demographic history of Estonia during the study period differs markedly from that of most Western European countries and the United States. Estonia was among the pioneers of the fertility transition in Europe, as a slow decline in crude death and birth rates occurred during the first half of the nineteenth century. The fertility decline in Estonia began with the generations born in the 1830s and 1840s; among cohorts born in the late 1890s, the mean number of children was close to two (Gortfelder & Puur, 2019). This decline persisted until the interwar period, so in the late 1920s fertility dropped below replacement for the first time in peacetime conditions (Klesment et al., 2010).

Among countries with comparable timing of demographic development, Estonia and Latvia were distinguished by the absence of a post-war baby boom. However, except for an increase in fertility, the other characteristics of a baby boom, such as an increase in marriages, a drop in ages for marriage and first birth and a decrease in childlessness, were present. Despite these developments, fertility remained below replacement, being the lowest in the world in 1950 (Katus et al., 2004).

Against the general trend in Europe, fertility in Estonia increased rather than decreased in the late 1960s, and by the late 1980s was higher than in any major region of the continent (Klesment et al., 2010). One of the reasons for this fertility increase was the housing shortage (primarily government-owned, controlled and distributed). Under these conditions, the prospects for obtaining an apartment for rent were substantially better for those young people who were married and had a child or children (Frejka & Sardon, 2004). Other factors favouring early and universal childbearing were pro-natalist ideology and policies. These policies were necessitated by a technologically lagging and labour-intensive, inefficient socialist economy, which generated a continuous demand for labour. Soviet-era living conditions included job security, low-cost housing, free education and health care, various entitlements associated with childbirth and childrearing, and highly structured career paths. The citizenry of Estonia, typically to state socialist countries, had grown accustomed to a relatively stable and predictable existence. However, standard living conditions were worse than in Western countries, and there were numerous disturbing concomitants to this lifestyle, such as curtailed civil liberties and shortages of everyday and long-lasting consumer goods (Frejka, 2017). Also, the atrocities of the Soviet regime of the 1940s and 1950s remained in the memories of contemporaries.

Despite these major differences, the social factors that are known to influence reproductive behaviour developed at similar or faster rates in Estonia as in many Western European countries. For instance, divorce rates had already started to rise during the interwar period (Sakkeus et al., 2016), equalling or exceeding those of Scandinavian countries from the 1970s onwards. Over the same period, non-married cohabitation began to prevail over marriage (Puur et al., 2016).

### 2.2. Data

Data for all Estonians born from 1905 to 1977 were requested from the Estonian Population Register in April 2023. Individuals in the last birth cohort were 45 years old at the time of data collection, and their offspring number was considered sufficiently close to lifetime reproductive success. The data comprised sex, birth years, the number of children born before 2022 and birth years for each person’s first and last child. Educational attainment data in the Population Register are automatically updated through linkages with other registries upon the occurrence of administrative events during which individuals are required to report their highest level of completed education.

Only data for individuals alive by the 15th year of age were used. Data for most persons also included self-reported educational attainment. Because educational data for the persons born at the beginning of the century were largely missing, we used the data from cohorts born from 1925 onwards for this study. The educational composition of the data set is shown in Supplementary Fig. S1 and the sample sizes in Table 1. As no sensitive personal data were involved, permission from the Research Ethics Committee was not required.

**Table 1.** Sample sizes for at least 15-year-old Estonians born between 1925 and 1977

Sample	Education	*N* women	*N* men
Number of individuals used for calculation of rLRS	Missing	71,463	92,847
	Primary	76,795	87,928
	Vocational	113,765	94,385
	Secondary	96,522	85,221
	Tertiary	104,773	64,217
Number of individuals for analyses of reproductive timing	Primary	67,167	64,647
	Vocational	104,096	81,583
	Secondary	86,450	69,690
	Tertiary	91,990	56,838

**Figure 1.**
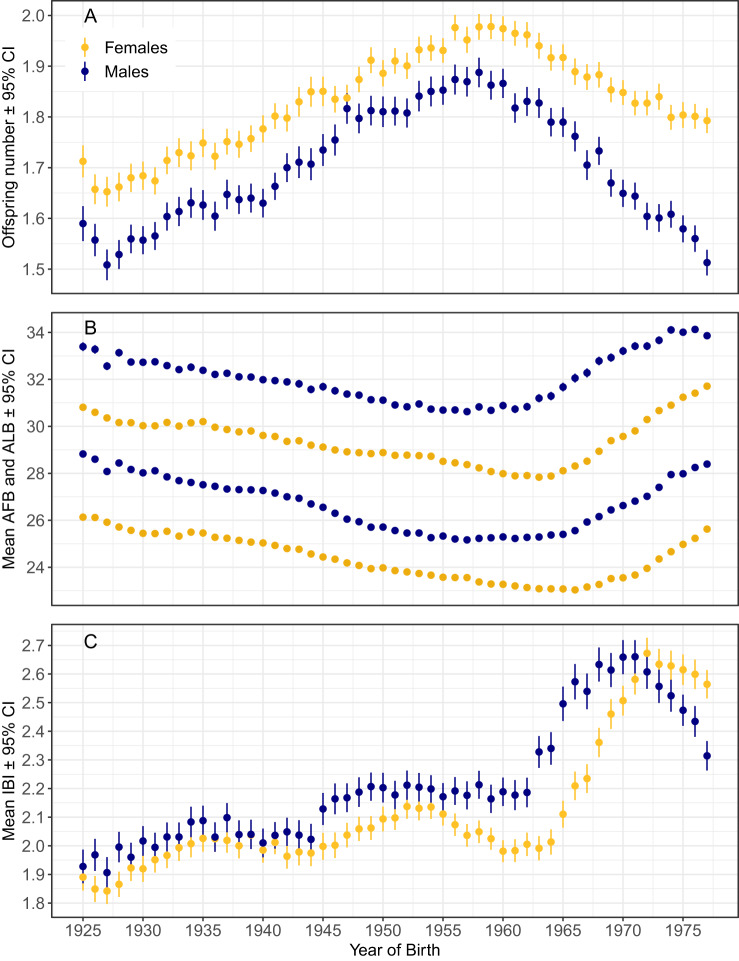
Birth cohort averages with 95% CI for offspring number, ages of first and last birth (AFB and ALB) and average interbirth intervals (IBIs) in years by sex. Sample sizes in Supplementary Table S1 and Supplementary Figure S1.

The parity data in the Population Register are more precise for women than men because the birth data are always linked to mothers’ identities, whereas fathers’ identities are linked only in cases where mothers report these. As a result, the average number of children for men was lower than that of women (Table 2, Fig. 1A). Incompleteness of parity data for men is a well-recognized problem in population studies and men have lower average reproductive success than women even in populations where the under-registration of fathers is low (see Jalovaara et al., 2022). We don’t know whether such under-reporting of fatherhood is systematically related to men’s education, but intuitively, one might expect that fatherhood was most likely unassigned to poorly educated men whose desirability in the mating market is lowest (Andersson et al., 2023). Such bias would mean that a number of (presumably poorly educated) fathers were misclassified as childless or that the actual number of children of such men was underestimated. Under this scenario, our calculations would overestimate both the strength of selection against poorly educated men and the strength of selection favouring well-educated men. However, our estimates of selection on reproductive timing are less biased because these include only men with children.

**Table 2.** Average offspring numbers by sex (M: male, F: female) and educational attainment for the whole study period (second column) and for the birth cohorts with maximum and minimum average offspring number for given sex/education category (third and fifth column)

Sample	1925−1977 mean, 95% CI, *N*	Birth cohort maximum mean, 95% CI, *N*	Birth cohort max. year	Birth cohort minimum mean, 95% CI, *N*	Birth cohort min. year
Total M	1.70 (1.70−1.71), 424,598	1.89 (1.86 − 1.92), 8342	1958	1.51 (1.48 − 1.54), 7280	1927
Total F	1.83 (1.83−1.84), 463,318	1.98 (1.95 − 2.00), 9397	1959	1.65 (1.62 − 1.68), 8697	1927
Primary M	1.64 (1.63−1.65), 87,928	1.79 (1.72 − 1.86), 1967	1947	1.20 (1.14 − 1.27), 1579	1975
Primary F	2.14 (2.13−2.15), 76,795	2.84 (2.66 − 3.01), 444	1959	1.91 (1.86 − 1.96), 3241	1926
Vocational M	1.91 (1.91−1.92), 94,385	2.15 (2.09–2.21), 1839	1954	1.72 (1.63–1.81), 552	1929
Vocational F	1.90 (1.90−1.91), 113,765	2.06 (2.02–2.10), 2889	1958	1.54 (1.46–1.61), 830	1927
Secondary M	1.73 (1.72−1.74), 85,221	1.91 (1.85–1.96), 1989	1953	1.45 (1.40–1.50), 2095	1977
Secondary F	1.87 (1.86−1.88), 96,522	2.11 (2.06–2.16), 2532	1960	1.48 (1.40–1.55), 911	1928
Tertiary M	1.98 (1.97−1.99), 64,217	2.16 (2.10–2.22), 1604	1960	1.61 (1.48–1.74), 276	1926
Tertiary F	1.74 (1.74–1.75), 104,773	1.85 (1.81–1.89), 2798	1961	1.23 (1.13–1.33), 410	1926

### 2.3. Statistical analyses

For measuring selection, we relied on the framework of Lande and Arnold (1983) as a standard approach for studying natural selection in contemporary human populations (e.g. Bolund et al., 2013; Hayward et al., 2015; Pettay et al., 2007; Stearns et al., 2012), including genome-wide association studies (e.g. Beauchamp, 2016; Sanjak et al., 2018). Relative lifetime reproductive success (rLRS) was calculated separately for men and women within each birth year (cohort) by dividing an individual’s offspring number by the cohort’s mean number of offspring. Cohort means were calculated, including individuals with unknown education. Standardized Lande–Arnold linear (β) and quadratic (γ) selection gradients (Lande & Arnold, 1983) for reproductive timing traits were estimated using linear and quadratic regressions of rLRS on cohort-standardized trait values (*z*-scores) for age at first birth (AFB), age at last birth (ALB), and average interbirth interval (IBI). Individual IBI values were calculated as the time span between the first and last birth (in years) divided by the total number of children. Estimates of quadratic selection gradients are double the quadratic regression coefficients. Selection gradients were calculated for different educational attainment levels over the whole 52-year study period (Fig. 4, Supplementary Table S1) and for each birth cohort separately (Fig. 5). Non-overlapping 95% confidence intervals are considered as indicative of statistically significant differences between group average trait values.

## 3. Results

### 3.1. Reproductive success and timing in the whole sample

The average offspring number (completed fertility rate) increased steadily from 1.5–1.7 among the 1927 birth cohort to 1.9–2 in the 1958 and 1959 cohorts, before beginning to decline in the birth cohorts of the 1960s (Fig. 1A; Table 2). Women’s ages of first and last births (AFB and ALB) declined up to the 1965 birth cohort, after which they began to rise. AFB and ALB of men showed parallel secular dynamics to these of women with an exception that AFB remained stable around 25 years of age among men born from 1954 to 1966 (Fig. 1B). Cohort average interbirth intervals (IBIs) fluctuated between 1.9 and 2.1 years until an abrupt increase occurred in the 1964 and 1965 birth cohorts, peaking with the cohort born in 1971 (Fig. 1C). In subsequent cohorts, IBIs began to decline again – particularly sharply among men, whose IBIs became shorter than those of women for the first time.

### 3.2. Reproductive success across educational levels

**Figure 2.**
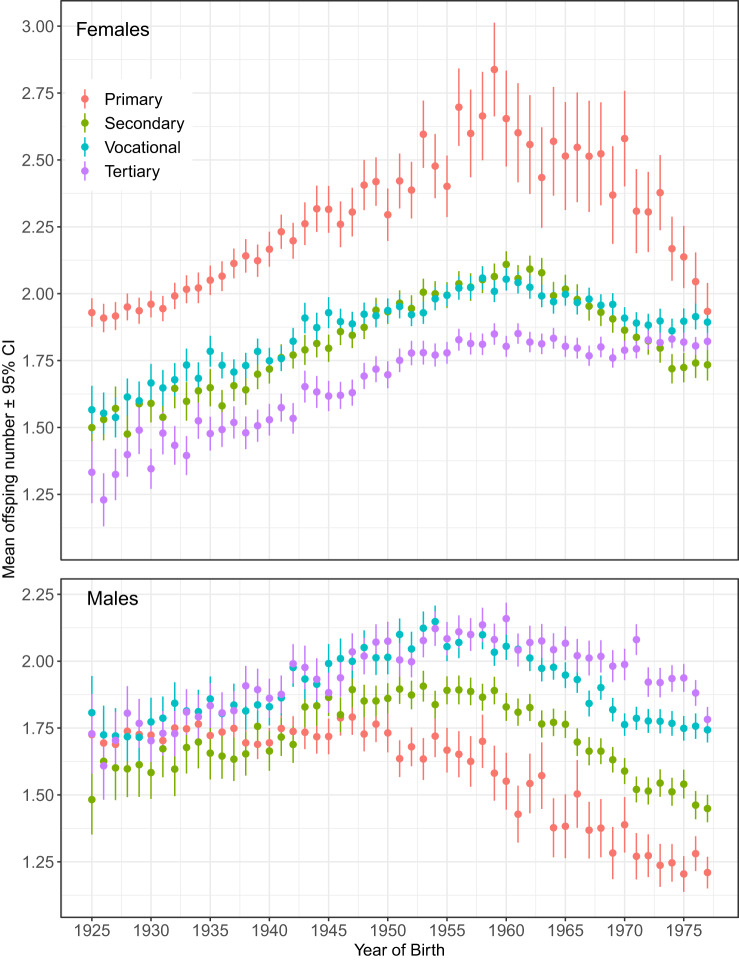
Birth cohort averages for offspring number with 95% CI across sexes and educational attainment levels. Sample sizes in Supplementary Table S1 and Supplementary Figure S1.

Women with primary education had the highest reproductive success throughout the study period (Fig. 2A, Table 2). Their average offspring number peaked at 2.8 in the 1959 birth cohort and remained around 2.5 children until 1970 birth cohort, after which it began to decline. In contrast, women with tertiary education followed a different temporal pattern: their average number of children increased from 1.23 in 1923 to 1.75 by the 1951 cohort and then remained nearly stable through the end of the study, peaking at 1.85 children in the birth cohort of 1961. On average, women with primary education had 0.4 more children than those with tertiary education. In the 1959 birth cohort, this difference neared one child (Fig. 2A, Table 2).

Birth rates among women with secondary and vocational education were generally similar and followed a trend parallel to that of women with primary education. However, a notable shift occurred in cohorts born from 1974 onward, when birth rates among women with secondary education dropped below those of all other educational groups.

Men’s reproductive rates followed different temporal patterns from women’s (Fig. 2B). Across educational groups, the number of offspring was generally similar among men born between 1925 and 1947. Starting with the 1950 birth cohort, however, the number of children fathered by men with primary education fell below that of all other educational groups. Men with secondary education showed a similar declining trend, with consistently lower reproductive rates than those with vocational or tertiary education. Men with tertiary and vocational education exhibited comparably high reproductive rates between the 1942 and 1962 cohorts. After this period, the reproductive success of men with tertiary education remained higher than that of any other men until the 1976 birth cohort.

Comparison of selection gradients over the entire study period revealed that selection on educational attainment was stronger for women than for men across all educational levels (Table 3). The difference was particularly pronounced for secondary education, where the ratio of selection gradients reached 4.5. This was primarily due to initially negative selection on secondary education among men born between 1925 and 1942 (Fig. 2B), which resulted in a low overall selection gradient for this education level (0.054) for men across the whole study period.

**Table 3.** Linear selection gradients (β) for educational attainment (all levels compared against individuals with primary education). Sample sizes for primary education: females 76,795; males 87,928

	Females			Males		
	β	95 % CI for β	*N*	β	95 % CI for β	*N*
Intercept	1.236			0.964		
Vocational	−0.226	−0.232, –0.220	113,765	0.169	0.161, 0.177	94,385
Secondary	−0.243	−0.249, –0.237	96,522	0.054	0.046, 0.062	85,221
Tertiary	−0.320	−0.328, –0.312	104,733	0.206	0.198, 0.214	64,217

### 3.3. Reproductive timing across educational levels

**Figure 3.**
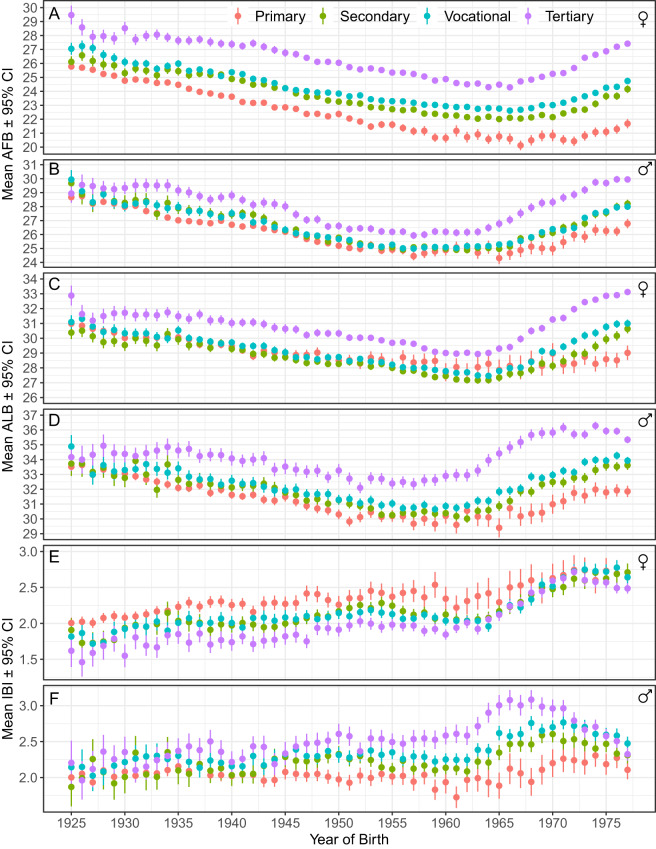
Birth cohort averages with 95% CI for offspring number, ages of first and last birth (AFB and ALB) and average interbirth intervals (IBIs) in years by educational attainment and sex. Sample sizes in Supplementary Table S1 and Supplementary Figure S1. See Supplementary Figures S2–S4 for fine-scale data.

Women with primary education started the reproduction earliest (e.g. around 20 years of age in 1963 birth cohort), followed by the women with secondary, vocational and tertiary education (Fig. 3A). The temporal trend in AFB was generally parallel across all educational strata with an exception that among the women with primary education, the rise of AFB did not start before the birth of 1973 cohort, and that the magnitude of delay in reproduction remained modest (1.3 years difference between birth cohorts of 1972 and 1977). In contrast, among the women with tertiary education, the AFB rise steadily from 24.4 to 27.4 years between birth cohorts of 1966 and 1977.

The AFB of men with different educational attainments fluctuated in parallel throughout the study period (Fig. 3B). The AFB of men with tertiary education was the latest compared to that of other men since the birth cohort of 1929. AFB of men with primary education was earlier than that of other educational strata in cohorts born between 1934 and 1941 and in cohorts born between 1965 and 1977. AFB of men with secondary and vocational education showed marked overlap throughout the study period.

The age of last reproduction showed generally similar time trends to the age of first reproduction. Women with tertiary education ended the reproduction latest during nearly the whole study period (Fig. 3C). Other educational groups did not differ much in their ALB before birth cohorts of the 1970s, when women with primary education ceased the reproduction first, followed by the women with secondary and vocational education.

ALB was the latest among men with tertiary education throughout the study period. At the same time, other educational categories did not clearly distinguish before the 1970s (Fig. 3D). Since the birth cohort of 1974, men with the lowest education ended the reproduction earliest, followed by the men with secondary and vocational education.

Interbirth intervals exhibited a reversed educational gradient across sexes: women with the lowest and men with the highest educational levels generally had the longest IBIs in most birth cohorts. Starting with the cohorts born in the 1950s, the educational gradient in IBIs became more pronounced in men than in women (Fig. 3E,F).

### 3.4. Natural selection on reproductive timing across educational levels

**Figure 4.**
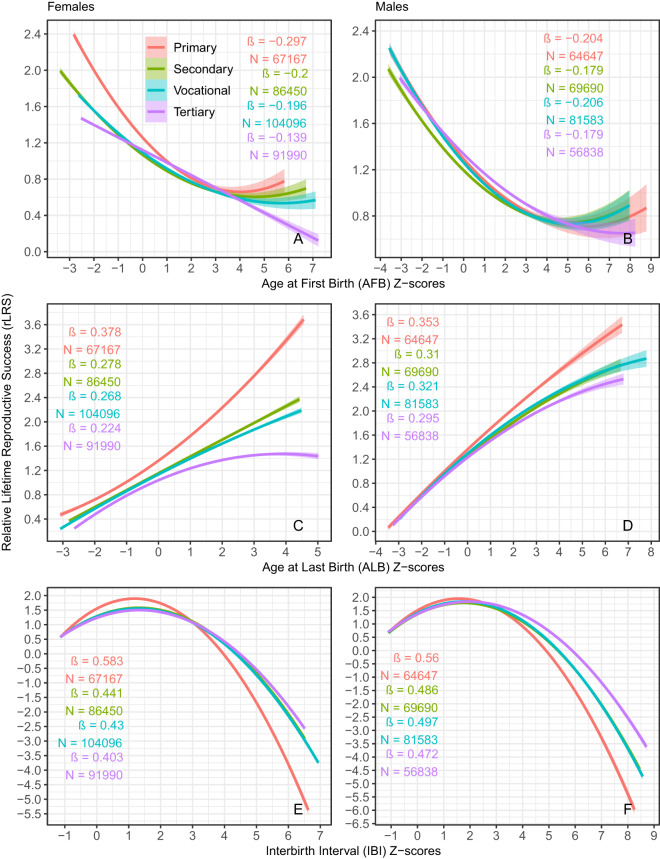
Quadradic selection curves with 95% CI for reproductive timing traits over the whole study period. ẞs are linear selection gradients (β_Q_) estimated from the quadratic regression model: rLRS = a + β_Q_(trait zscore) + 1/2 γ(trait zscore)^2^ + ε. See Supplementary Figure S5 for selection curves and gradients for unstandardized trait values and Table S2 for statistics.

**Figure 5.**
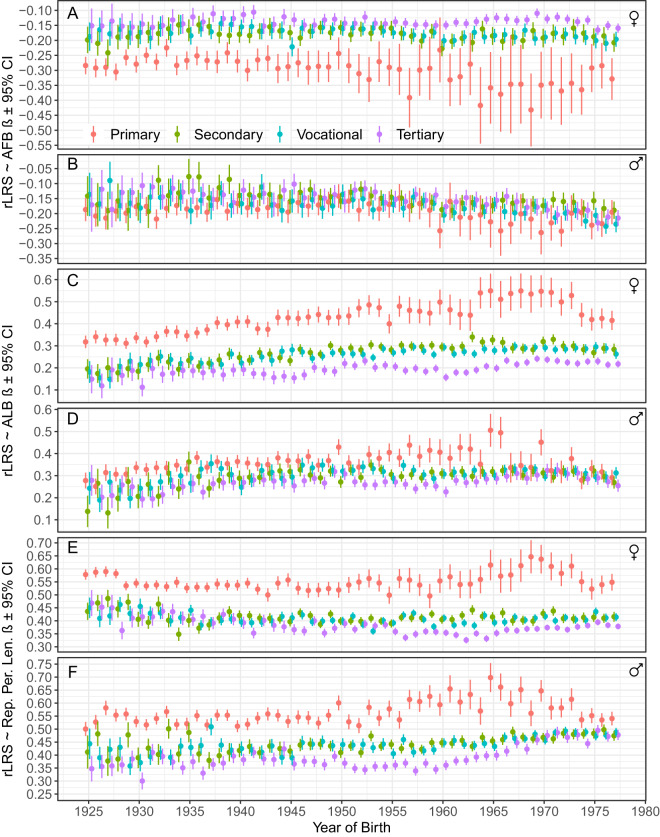
Cohort average linear selection gradients (ẞ and 95% CI) on reproductive timing traits by sex and educational attainment levels. See Supplementary Figures S6–S8 for fine-scale data.

Natural selection for earlier reproduction in women was almost entirely linear across all educational levels (Fig. 4A, Supplementary Table S1). The linear selection gradient was approximately twice as strong among women with primary education (β = − 0.30) compared to those with tertiary education (β = − 0.14. Linear selection gradients for AFB for women with secondary and vocational education were intermediate and identical (β = − 0.20). Linear selection gradients among women with education above the primary level remained relatively stable throughout the study period, whereas selection for earlier reproduction intensified in cohorts born between 1964 and 1974 among women with primary education (Fig. 5A).

Linear selection gradients for AFB in men showed considerably less variation across educational strata than in women: β = − 0.21 for vocational, − 0.20 for primary and − 0.18 for secondary and tertiary education (Fig. 4B, Fig. 5B, Supplementary Table S1). The within-cohort overlap of selection gradients across educational levels was also greater in men than women.

The strength of natural selection on ALB was greater than that on AFB (Fig. 4, Fig. 5A–D; Supplementary Table S1). Again, the selection was nearly entirely linear and nearly twice as strong among women with primary education (β = 0.38) compared to those with tertiary education (β = 0.22). Linear selection gradients for ALB for women with secondary (β = 0.28) and vocational (β = 0.27) education were intermediate and close to each other (Fig. 4C, Fig. 5C, Supplementary Table S1). Selection gradients among women with primary education increased more steeply across birth cohorts from 1925 to 1973 than those of women with higher education levels (Fig. 5C).

Linear selection gradients for ALB in men varied between 0.30 (tertiary education) and 0.35 (primary education). Strength of selection for ALB in men with vocational (β = 0.32) and secondary (β = 0.31) education was intermediate (Fig. 4, Fig. 5D, Supplementary Table S1). Again, within-cohort overlap of selection gradients across educational levels was greater in men than in women (Fig. 5 CD).

In contrast to the predominantly linear selection on AFB and ALB, selection on the pace of reproduction was stabilizing (Fig. 4E,F, Supplementary Table S1). The magnitude of the quadratic selection gradients (γ) for IBIs ranged from −0.24 to −0.50, approximately an order of magnitude greater than the range observed for AFB and ALB, which varied between −0.06 and 0.08 (Supplementary Table S1). Among women with primary education, selection favoured those with average interbirth intervals between 1 and 1.5 standard deviations (SD) (Fig. 4E). For women with higher levels of education, fitness curves for IBIs were nearly identical, with peak values around 1.5 SD. The fitness curves for women with primary and above-primary education intersected at approximately 3 SD, indicating that women with primary education experienced a steeper decline in fitness at longer interbirth intervals compared to their more educated counterparts.

The reproductive success of men across different educational levels increased similarly up to IBI values between 1 and 1.5 SD units, after which the fitness curves for men with primary, vocational/secondary and tertiary education diverged (Fig. 5F). At longer IBIs, selection favoured slower reproduction in men with tertiary education compared to those with primary education. For men with secondary and vocational education, the fitness benefits of slower reproduction were intermediate.

## 4. Discussion

### 4.1. Population-level demographic patterns

The temporal trend in cohort fertility rates (average number of offspring per birth year, Fig. 1A) aligns with previous Estonian estimates based on census (Klesment et al., 2010) and survey (Katus, 2000) data. The pattern of peaking and subsequent decline in birth rates among cohorts born around 1960 differs from that observed in countries that experienced a post–World War II baby boom (Katus et al., 2004). The absence of a post-war baby boom in Estonia has been attributed to population loss, political violence and the lack of post-war economic improvement (Gortfelder et al., 2025).

Secular trends in the timing of first births, too, were consistent with previously published data for the Estonian population (Katus & Puur, 2006; Nugin et al., 2016). The pattern of advancement – and subsequent delay – of AFB up to around the 1965 birth cohort (Fig. 1B) is similar to trends observed in Greece, the Czech Republic, Hungary and Slovenia. In Nordic countries, postponements of first births continued in cohorts born after 1970 (Hellstrand et al., 2021). In contrast, in several other European countries and the United States, the turning points to delay reproduction in women occurred earlier, among cohorts born between the 1930s and 1950s (Beaujouan et al., 2023; Frejka & Sardon, 2006).

One of the factors that sustained early family formation and childbearing in Estonia up to the cohorts born after 1965 may have been the housing allocation system under the state socialist regime. New dwellings were typically distributed according to administrative regulations, and to become eligible for housing, a couple generally had to live in conditions that fell below a certain minimum standard, particularly in terms of per-capita floor space. In this context, the birth of a child increased young couples’ chances of advancing in the housing queue. The persistence of these mechanisms until the late 1980s likely explains why the shift towards postponing childbearing occurred relatively late in Estonia (Klesment et al., 2010).

Despite the largely parallel trends in AFB and ALB, the reproductive pace increased among both men and women from the 1920s cohorts through to the 1970 birth cohort. In the following birth cohorts, IBIs started to decline. The decline was particularly abrupt among men, which is likely attributable to increased under-registration of fatherhood among those born in 1970s (see Fig. 1A).

The gender gap in cohort average offspring number has been increasing most rapidly among parents with lowest educational level throughout the study (Fig. 2). This pattern indirectly supports the idea that men with the lowest education prevailed among the unregistered fathers. Such interpretation would be also consistent with findings that the prevalence of single motherhood increased with declining stigma against out-of-wedlock births, growing female economic independence and shifting norms about marriage, cohabitation and divorce (e.g. Kutsar et al., 2012; Schnor & Jalovaara, 2020).

### 4.2. Sexually antagonistic selection on educational attainment

Over the whole study period, women with primary education had an average of 2.14 children, which is 0.4 more than the average of 1.74 children among women with tertiary education (Fig. 2A, Table 2). This difference is nearly twice as large as the 0.22-child gap observed between the highest and lowest educational attainment groups in the 1958 British National Child Development Study cohort (Nettle & Pollet, 2008). Among the birth cohorts with the highest (1959) and lowest (1926) fertility, the difference in the number of children between the most- and least-educated women was even more pronounced – 1 child and 0.68 children, respectively (Table 2).

The educational gradient for reproductive success in women retained its direction throughout the 52-year study period, although the magnitude of selection gradients at different educational levels varied considerably. Women’s reproductive rates peaked among cohorts born from 1959 to 1962, during which the average offspring number exceeded 2 in all educational groups except those with tertiary education. These findings are consistent with 2011 population census data, showing that throughout the twentieth century, Estonian women with primary education bore 0.5–0.75 more children on average than women with tertiary education (Tiit, 2013). Educational gradient in offspring number is consistent with nearly universal findings that reproductive performance in women associates negatively with educational attainment (Beaujouan et al., 2023; Berrington et al., 2015; Hellstrand et al., 2024; Kirk et al., 2001; Nisén et al., 2021; Sobotka, 2017; Van Bavel et al., 2018). The convergence of reproductive rates among women of all educational levels in the birth cohorts of the 1970s mirrors trends observed in Denmark, Norway and Sweden. In all these countries, this convergence was caused by the decline of reproductive rates among the least educated, while the reproductive rates of the most educated women either increased or stabilized. In contrast, women with the lowest educational attainment in Finland maintained a reproductive advantage until the birth cohorts of the 1970s (Jalovaara et al., 2019). The reproductive advantage of the lowest-educated women over all other educational groups was also retained in the Czech Republic through birth cohorts from 1900 to 1970 (Zeman, 2018).

In contrast to women, men with tertiary education had the highest average offspring number (1.98), wheras those with primary education had the lowest (1.64; Table 2), followed by men with secondary (1.73) and vocational education (1.91; Table 2). The difference in the number of offspring between men with the highest and lowest levels of education (0.34 children) was smaller than that observed among women, but still 3.8 times larger than the 0.09-child-gap observed in the 1958 British birth cohort (Nettle & Pollet, 2008). Notably, the educational differences in male reproduction in our study were less clearly distinguishable than in women until the 1950s birth cohorts (Fig. 2). Sexual antagonism in selection on educational attainment thus became distinctly evident in cohorts born from the mid-twentieth century onward. It should be recalled, however, that our analyses likely overestimate the strength of selection against poorly educated men, particularly in the later-born cohorts, which would eventually somewhat weaken the sexual antagonism in selection on educational attainment.

The increasing educational gradient in male reproductive success among cohorts born since the 1950s can likely be attributed to the growing value of male education in the mating market since the 1990s. During the Soviet era, monetary returns on education were minimal: in 1989, university-educated employees earned only 8% more than the economy-wide average wage, and workers with only primary education earned just 3% less than the average (Noorkõiv et al., 1998). In contrast, the transition to a market economy dramatically increased the returns from education. Men with primary education experienced disproportionately large income losses and higher unemployment rates during the 1990s (Noorkõiv et al., 1998). This effect of paternal education had tangible consequences on offspring outcomes. For example, among a subset of Estonian schoolchildren born between 1980 and 1987, those with fathers who had primary education were shorter, had smaller heads and reported lower resource availability compared to children of fathers with education beyond the primary level (Lauringson et al., 2020). Notably, these fathers were, on average, 3.3 cm shorter than more highly educated fathers (see also Valge et al., 2019).

Reports of positive associations between educational attainment and reproductive success in contemporary men are less consistent than those for women (reviewed by Nisén et al., 2013), possibly due to men being understudied in this regard (Kravdal, 2007). In Nordic countries, the fertility of low-educated men has consistently been the lowest through birth cohorts from 1940 to 1969 (Jalovaara et al., 2019; Nisén et al., 2014). Similar to the current study, the gap between low-educated men and other educational groups of men has widened somewhat in Denmark, Finland and Norway since the 1950s (Jalovaara et al., 2019). Outside the Nordic countries, the association between education and fertility in men varies from positive through flat to negative (reviewed by Nisén et al., 2014).

The detection of sexually antagonistic selection on educational attainment in this study confirms previous findings from a sample of Estonian schoolchildren (15,253 girls and 11,842 boys) born between 1937 and 1962 (Valge et al., 2022). Sexually antagonistic selection on educational attainment is consistent with several previous studies based on population registries (reviewed by Jalovaara et al., 2019; Valge et al., 2022). For example, opposing education–fertility gradients for men and women were observed in registry data for Norwegian cohorts born between 1940 and 1964 (Kravdal & Rindfuss, 2008). A similar pattern was found in a study of Finnish twins born between 1950 and 1957 (Nisén et al., 2013), as well as in a large register-based analysis of birth cohorts from 1940 to 1974 across four Nordic countries (Jalovaara et al., 2019). Sexually antagonistic education–fertility gradients have also been reported in the US General Social Survey (Hopcroft, 2006), the Framingham Heart Study (Stearns et al., 2012) and Swedish population register data (Fieder & Huber, 2007).

However, the Wisconsin Longitudinal Study in the US found that although educational attainment was phenotypically negatively associated with the number of children in both sexes, genetic predisposition for education negatively affected reproductive success only in men (Fieder & Huber, 2022). Negative genetic correlations between educational attainment and LRS have also been reported in the UK Biobank (Hugh-Jones & Abdellaoui, 2022; Warrington et al., 2021). Similar to the present study, selection against educational attainment was stronger in women than men. Selection against genetic variants associated with higher educational attainment was also reported in a meta-analysis of 62 cohorts of European ancestry (Barban et al., 2016). Finally, negative selection against polygenic scores for education – along with evidence of evolutionary response to this selection, which was weaker in males than in females – has been documented in 129,808 Icelanders born between 1910 and 1990 (Kong et al., 2017).

Direct selection gradients on educational attainment in our study ranged from −0.32 to 0.21 (Table 3), exceeding those reported in the UK Biobank (−0.061 for women, –0.009 for men) (Sanjak et al., 2018) and US Health and Retirement Study (−0.057 for women, −0.022 for men) (Beauchamp, 2016). However, our estimates on men were comparable in magnitude to selection gradients on male wealth and income reported in three industrial societies (β = 0.10–0.17; Nettle & Pollet, 2008). In contrast, marrying a man from a ‘rich’ social class in pre-industrial Finland was associated with a selection gradient of −0.17, compared to the absence of selection when married to ‘poor’ men (Hayward et al., 2015).

### 4.3. Reproductive timing across sexes and educational levels

Differences in reproductive timing across educational levels were considerably larger than the annual fluctuations over the generations studied (Figs. 1 and 3). Temporal trends in the ages of first and last birth were generally parallel across all educational strata and sexes (Fig. 3). The educational differences in AFB were relatively stable until the birth cohort of 1965, after which they started to increase among both men and women (Fig. 3A). These patterns differ from the Norwegian data where difference in AFB between the women with lowest and highest education showed steady increase from birth cohorts of 1940 (5.6 years) to 1964 (7.5 years) (Kravdal & Rindfuss, 2008). In Estonia, the corresponding differences were 3.8 and 3.7 years. The educational gap in age at first birth peaked at 5.7 years in the 1977 birth cohort, largely driven by women with tertiary education, whose AFB rose faster than that of women with primary education.

As the vast majority of the studied parents began childbearing during the Soviet era, the relatively small educational gap in AFB up to the 1965 birth cohort may reflect the specific features of state socialist economies – namely, low labour market returns to education, state-guaranteed full employment with highly structured career paths, and extensive public childcare provision. These conditions likely resulted in relatively low and similar opportunity costs of childbearing for women across different educational levels (Klesment & Puur, 2010). In line with this reasoning, the impact of educational attainment on AFB was less strong in East Germany than West Germany (Kreyenfeld, 2000).

Among men, educational differences in AFB were less pronounced but still notable. The consistent pattern of later AFB among tertiary-educated men since the 1929 cohort parallels that of women, suggesting that the long-standing effects of extended education on reproductive timing have operated similarly in both sexes. Comparable results were found in a study of six European countries in birth cohorts of 1960–1987 (Trimarchi & Van Bavel, 2018) and in Finnish birth cohorts of 1940–1950 (Nisén et al., 2014). These findings also show that an early start to reproduction was not necessary for achieving a high offspring number among men with tertiary education. In contrast to women, cohort-average ages at first birth largely overlapped among men with primary, secondary and vocational education until the 1970s birth cohorts. This indicates that, for men, educational differences below the tertiary level had little effect on the timing of reproduction.

Both men and women with tertiary education ended reproduction later than their less educated peers, and secular trends largely mirrored those observed in AFB. The result that highly educated women ceased reproduction later than others aligns with a few previous studies on ALB (Singh et al., 2023 and references therein; Varas Enríquez et al., 2022). The most straightforward explanation is likely the postponement of childbearing until after graduation (e.g. Mattison et al., 2018). For highly educated men, a further reason for delayed cessation of reproduction may be their prolonged accumulation of social capital and other resources, which ultimately attract women with high and sustained reproductive potential (Buunk et al., 2002; Fales et al., 2016). Additionally, such resourceful men may extend their reproductive careers by repartnering with younger spouses, as higher educational attainment can increase the likelihood of forming multiple partnerships (De Graaf & Kalmijn, 2003; Maslauskaitė & Baublytė, 2015; Nisén et al., 2014; Prskawetz et al., 2003; Schnor et al., 2017; but see Jalovaara et al., 2022; Kolk & Barclay, 2019; Lappegård et al., 2011).

Unlike AFB and ALB, the pace of reproduction exhibited a sexually antagonistic educational gradient: women with the highest education and men with the lowest education had the shortest interbirth intervals. The association between higher education and shorter birth spacing among women has been previously documented in several northern, western and southern European countries (Klesment et al., 2014). This pattern aligns with the commonly observed trend that women of higher socioeconomic status tend to exercise greater control over their reproductive behaviour (Varas Enríquez et al., 2022). Short birth intervals of highly educated women have been explained by a ‘time squeeze’ effect, arising from postponement of entry to motherhood, thereby reducing birth intervals to meet fertility goals, or to minimize the loss of wages, and job market opportunities, and reduce the time spent away from work in childcare (Bartus et al., 2013; Compans et al., 2023; Klesment et al., 2014; Rendall et al., 2020).

We are aware of two studies that have analysed the IBIs of men concerning educational levels. In Finnish birth cohorts of 1940–1950, men with tertiary education had the fastest reproductive pace, measured as the interquartile range of age at having children (Nisén et al., 2014). Among Finnish men born between 1955 and 1975, interbirth intervals of both men and women mostly decreased with increasing educational levels (but this effect was sex-specifically mediated by AFB; Berg & Rotkirch, 2014). Our finding that, unlike women, more highly educated men had the most extended interbirth intervals contrasts with the results of both Finnish studies cited above. This indicates that, in addition to sex, the mechanisms linking parental education to the pace of reproduction may vary even between neighbouring countries.

### 4.4. Natural selection on reproductive timing across educational levels

The linear selection gradient on women’s age at first birth among women with primary education (β = −0.30) was similar to that observed over all women of the UK Biobank (Sanjak et al., 2018). Compared to those reported in pre-industrial societies (Bolund et al., 2013; Bürkli & Postma, 2014; Käär et al., 1996; Milot et al., 2011; Pettay et al., 2007), selection gradients on components of reproductive timing in our study were similar in magnitude and direction, though slightly weaker (β = −0.30–0.58; Fig. 4, Supplementary Table S1).

Among women, the strength of natural selection on all components of reproductive timing varied across educational levels. In men, selection on AFB showed little variation by education, whereas selection on ALB and IBIs was strongest among those with the lowest educational attainment. Natural selection on reproductive timing was thus generally strongest among parents with the lowest education and weakest among parents with the highest education. Among individuals with secondary and vocational education, the strength of selection was intermediate and, in most cases, of similar magnitude.

Beyond sexually antagonistic selection, empirical evidence that natural selection acts at varying rates across subgroups within modern populations remains limited. Our findings align best with a study in the UK Biobank (Hugh-Jones & Abdellaoui, 2022) showing that natural selection effects on polygenic scores of anthropometric and behavioural traits were stronger in groups with lower income and education, younger parenthood, not living with a partner and more lifetime sexual partners. Outside these groups, the effects of natural selection were weaker and often statistically insignificant. Among women with AFB above 22 years, the direction of selection was reversed. In the US Health and Retirement Study, natural selection on the polygenic scores of health traits was stronger for low-income groups and unmarried respondents (Hugh-Jones & Edwards, 2024). However, unlike the UK Biobank study, they found little evidence for stronger natural selection among people with lower education.

Hugh-Jones and Abdellaoui (2022) and Hugh-Jones and Edwards (2024) interpret their findings in the context of the economic theory of fertility (Becker, 1960). The theory assumes that parents make rational decisions about how many children to have and how much to invest in them, balancing the economic costs and benefits. It states that higher potential earnings have two opposite effects on fertility: a fertility-increasing income effect (higher income makes children more affordable) and a fertility-lowering substitution effect (time spent on childrearing has a higher cost in foregone earnings). Hugh-Jones and Abdellaoui (2022) suggest that the negative association between income and education (and traits related to these) versus fertility indicates that substitution effects dominate income effects, explaining why rising income or education levels typically shift preferences towards fewer, ‘higher-quality’ children.

Historical evidence for variation in the intensity of natural selection across population subgroups comes from pre-industrial Finland. Pettay et al. (2007) found the strongest selection on earlier age at first reproduction in women of the poorest wealth class, whereas selection favoured older age at reproductive cessation among the wealthy and middle-class mothers. The authors interpret their findings in the context of the theory of life-history evolution, which predicts stronger selection for early reproduction under conditions of high mortality and a lower chance of reproducing later in life, as was the case for the poorer women. However, even though the poor women would have benefited the most from early reproduction, they had the latest mean AFB. The authors suggest that poor women might have delayed their reproduction through behavioural means to adjust their child number to match their unfavourable economic circumstances.

The situation in the present study differs in that women with the lowest levels of education – used here as a proxy for the lowest social class – exhibited both the strongest selection for earlier reproduction and, typically to developed societies, the earliest average AFB throughout the study period. Notably, such women (along with the least-educated men) also displayed the strongest selection for the age of last birth. However, both women and, in particular, men with only primary education were also the first to cease reproduction compared to better-educated parents. Stabilizing selection for intermediate IBIs also showed the strongest quadratic selection gradients among minimally educated parents of both sexes (Fig. 4, Supplementary Table S1). At that, men with primary education exhibited the fastest, while women with primary education showed the slowest reproductive pace for most of the study period (Fig. 3).

Altogether, these results indicate that variation in reproductive timing across societal strata does not consistently reflect the selective pressures acting on recent generations. This pattern echoes findings from a study of pre-industrial Finns spanning up to 12 generations, where age at first birth remained relatively high over 350 years, despite strong selection for earlier reproduction and strong genetic correlations with fitness (Bolund et al., 2013).

It is important to note, however, that the traits considered here differ in how selection was measured. Educational attainment is defined for all individuals, including those who never reproduced, enabling standard approach to measuring selection (covariance between trait values and reproductive success). On the other hand, parameters of timing of reproduction (AFB, ALB, IBI) are only defined for parous individuals. Thus, our estimates of selection on reproductive timing describe variation among those who reproduced, but do not capture selection acting through childlessness. If the probability of remaining childless covaries with reproductive timing, our estimates may underestimate the total strength of selection at the population level. Such a scenario is likely, given that, genome-wide association studies have shown that genetic variants associated with later AFB predicted higher chances for remaining childless, especially among women (Verweij et al., 2019).

Apparent absence of response to selection for heritable life-history traits demonstrates that cultural, economic and social influences can override natural selection (Courtiol et al., 2016; Tropf, Stulp, et al., 2015). For example, although natural selection strongly favours early reproduction in women, Europe and the United States saw a marked delay in the age of first birth during the latter half of the twentieth century. Likewise, the widespread expansion of female education in developed countries continues, despite natural selection favouring women with lower levels of schooling (Courtiol et al., 2016; Tropf, Stulp, et al., 2015). An example of how phenotypic selection can obscure or override genotypic selection is provided by the study of Kong et al. (2017). Among Icelandic men, genetic variants associated with higher educational attainment were selected against, even though higher educational attainment itself was not associated with reduced fertility.

This study provides further evidence that the course of human evolution in modern societies is shaped by conflicting and inconsistent selective pressures. Our findings and those from pre-industrial Finland (Bolund et al., 2013) indicate that at the phenotypic level, moderately heritable reproductive traits can remain resistant to the forces of natural selection over generations. These findings indicate that, although natural selection can be clearly detected, predictions regarding its demographic consequences and phenotypic responses in human populations remain speculative.

On the other hand, evolutionary changes can sometimes occur rapidly, as demonstrated by a study from Iceland, showing that selection against genetic variants associated with educational attainment can lead to measurable change in the genetic composition of a population in a few generations (Kong et al., 2017). The same study also revealed that these genetic variants directly affected reproductive success, independent of the actual level of education attained. A response to selection can just remain unnoticed at the phenotypic level even if the genomic response has occurred.

Given that the strength of selection varies across sexes and educational groups, it has also been proposed that such dynamics may contribute to increasing inequality in polygenic scores (Hugh-Jones & Abdellaoui, 2022). As those authors emphasize, patterns of natural selection are not the outcome of a single, society-wide phenomenon. Instead, they result from opposing forces, operating in different parts of society and pulling in different directions. This highlights the need for future research to compare selection processes across various societal subgroups – ideally incorporating both phenotypic and genetic perspectives simultaneously, and accounting for the potential role of sexual antagonism.

## Supporting information

Meitern and Hõrak supplementary material Meitern and Hõrak supplementary material

## Data Availability

The R code is available at https://doi.org/10.5281/zenodo.15704019

## Supplementary material

The supplementary material for this article can be found at https://doi.org/10.1017/ehs.2025.10019.

## Research transparency and reproducibility

The data used in this study were obtained from Estonian Population Register. As no sensitive personal data were involved, permission from the Research Ethics Committee was not required. The original data, along with the R code, are available at https://doi.org/10.5281/zenodo.15704019
