# Supplementary material for: Natural selection on reproductive timing varies by education in twentieth-century Estonia
Source: Evol Hum Sci. 2025 Sep 8;7:e33. doi: 10.1017/ehs.2025.10019 (PMC12645315; doi:10.1017/ehs.2025.10019)
Supplement: Meitern and Hõrak supplementary material [file S2513843X25100194sup001.pdf]

Electronic Supplementary Material

Table S1. Standardized Lande–Arnold linear (β, β_Q) and quadratic (γ) selection gradients for reproductive timing traits, estimated using linear and quadratic regressions of rLRS on cohort-standardized trait values (z-scores) for age at first birth (AFB), age at last birth (ALB), and average interbirth interval (IBI) for the whole study period. Individual IBI values were calculated as the time span between the first and last birth (in years) divided by the total number of children. Estimates of γ and corresponding standard errors (SE) are doubled quadratic regression coefficients and their SEs. M: males, F: females.

Trait	Sex	Education	β	SE (β)	β_Q<br>γ	SE (β_Q)<br>SE (γ)	N
**AFB**	**F**	Primary	-0.278	0.003	-0.297	0.007	67,167
					0.074	0.002	
		Secondary	-0.180	0.002	-0.200	0.005	86,450
					0.043	0.002	
		Vocational	-0.176	0.002	-0.196	0.004	104,096
					0.034	0.002	
		Tertiary	-0.138	0.001	-0.139	0.004	91,990
					0.000	0.003	
	**M**	Primary	-0.185	0.003	-0.204	0.006	64,647
					0.035	0.003	
		Secondary	-0.156	0.002	-0.179	0.006	69,690
					0.035	0.003	
		Vocational	-0.181	0.002	-0.206	0.005	81,583
					0.040	0.003	
		Tertiary	-0.157	0.002	-0.179	0.006	56,838
					0.024	0.003	
**ALB**	**F**	Primary	0.385	0.003	0.378	0.005	67,167
					0.058	0.002	
		Secondary	0.277	0.002	0.278	0.003	86,450
					-0.001	0.002	
		Vocational	0.266	0.002	0.268	0.003	104,096
					-0.015	0.002	
		Tertiary	0.203	0.001	0.224	0.003	91,990
					-0.058	0.003	
	**M**	Primary	0.350	0.003	0.353	0.006	64,647
					-0.014	0.002	
		Secondary	0.302	0.002	0.310	0.004	69,690
					-0.025	0.002	
		Vocational	0.309	0.002	0.321	0.004	81,583
					-0.030	0.003	
		Tertiary	0.273	0.002	0.295	0.006	56,838
					-0.031	0.003	
**IBI**	**F**	Primary	0.343	0.003	0.583	0.006	67,167
					-0.492	0.002	
		Secondary	0.279	0.002	0.441	0.003	86,450
					-0.334	0.002	
		Vocational	0.277	0.001	0.430	0.003	104,096
					-0.333	0.001	
		Tertiary	0.271	0.001	0.403	0.003	91,990
					-0.303	0.003	
	**M**	Primary	0.377	0.003	0.560	0.006	64,647

					-0.356	0.002	
		Secondary	0.317	0.002	0.486	0.004	69,690
					-0.278	0.002	
		Vocational	0.312	0.002	0.497	0.004	81,583
					-0.283	0.002	
		Tertiary	0.286	0.002	0.472	0.005	56,838
					-0.240	0.009	

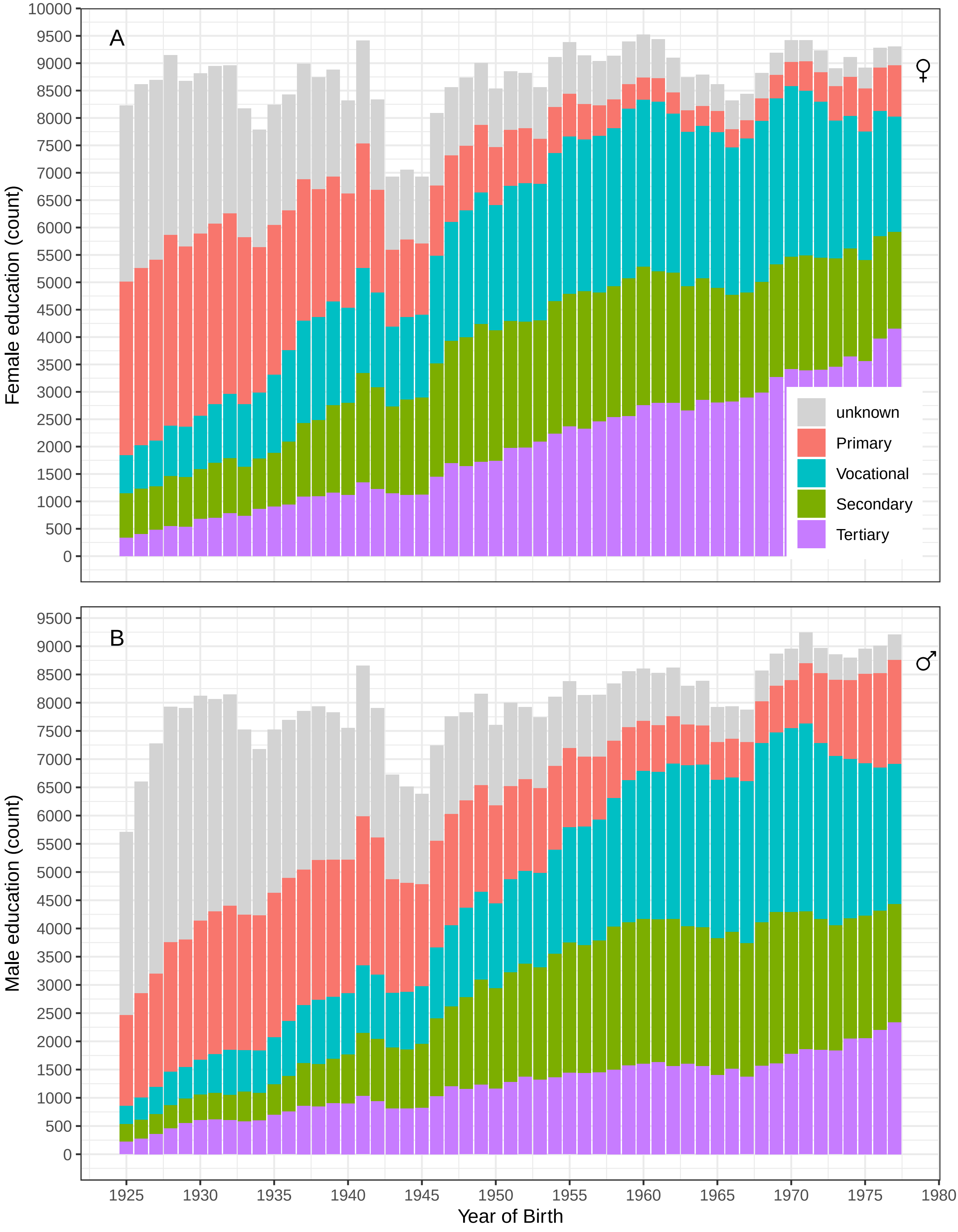

Figure S1. Sample sizes by birth cohort, education and sex.

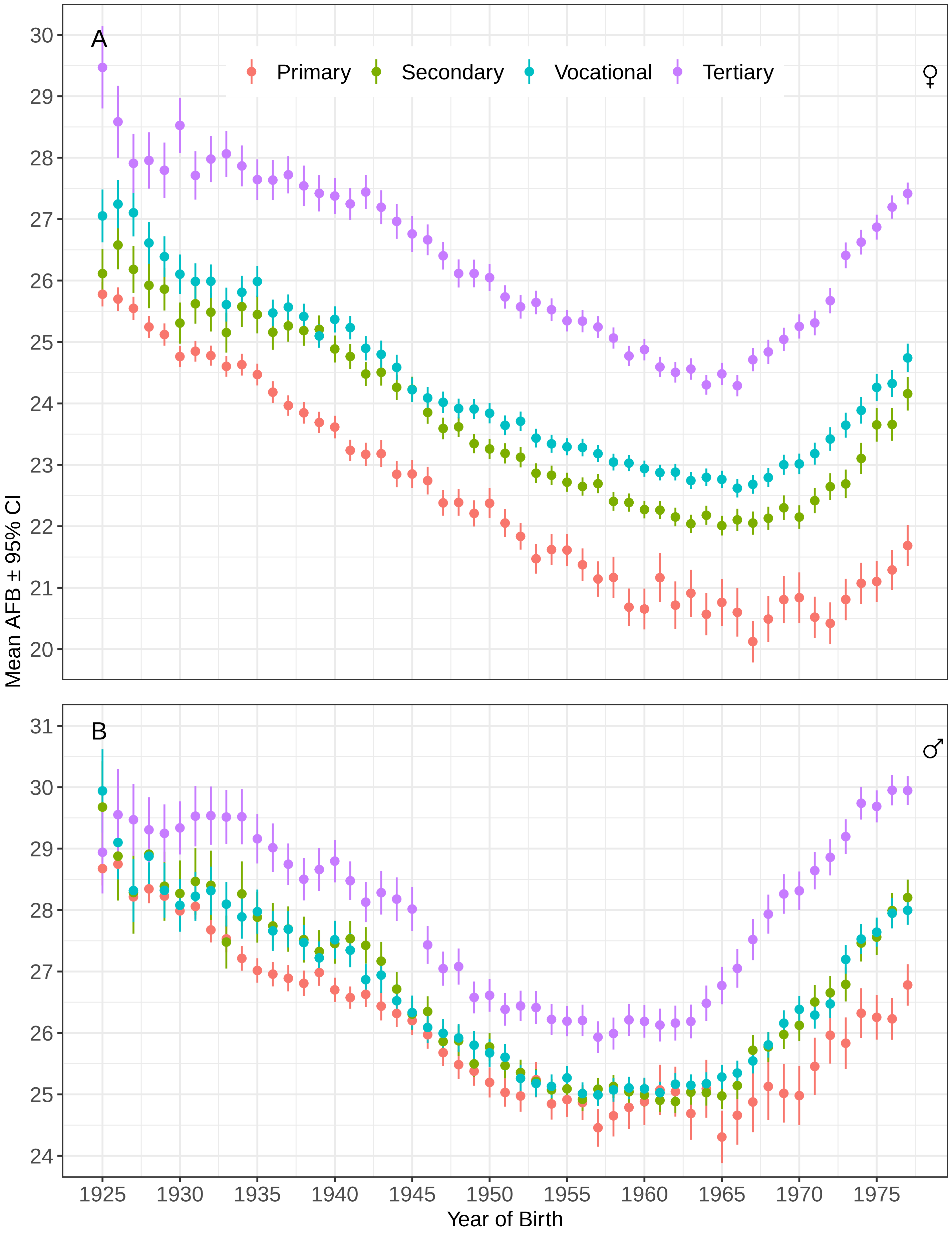

Figure S2. Birth cohort averages with 95% CI for age of first birth (AFB, years).

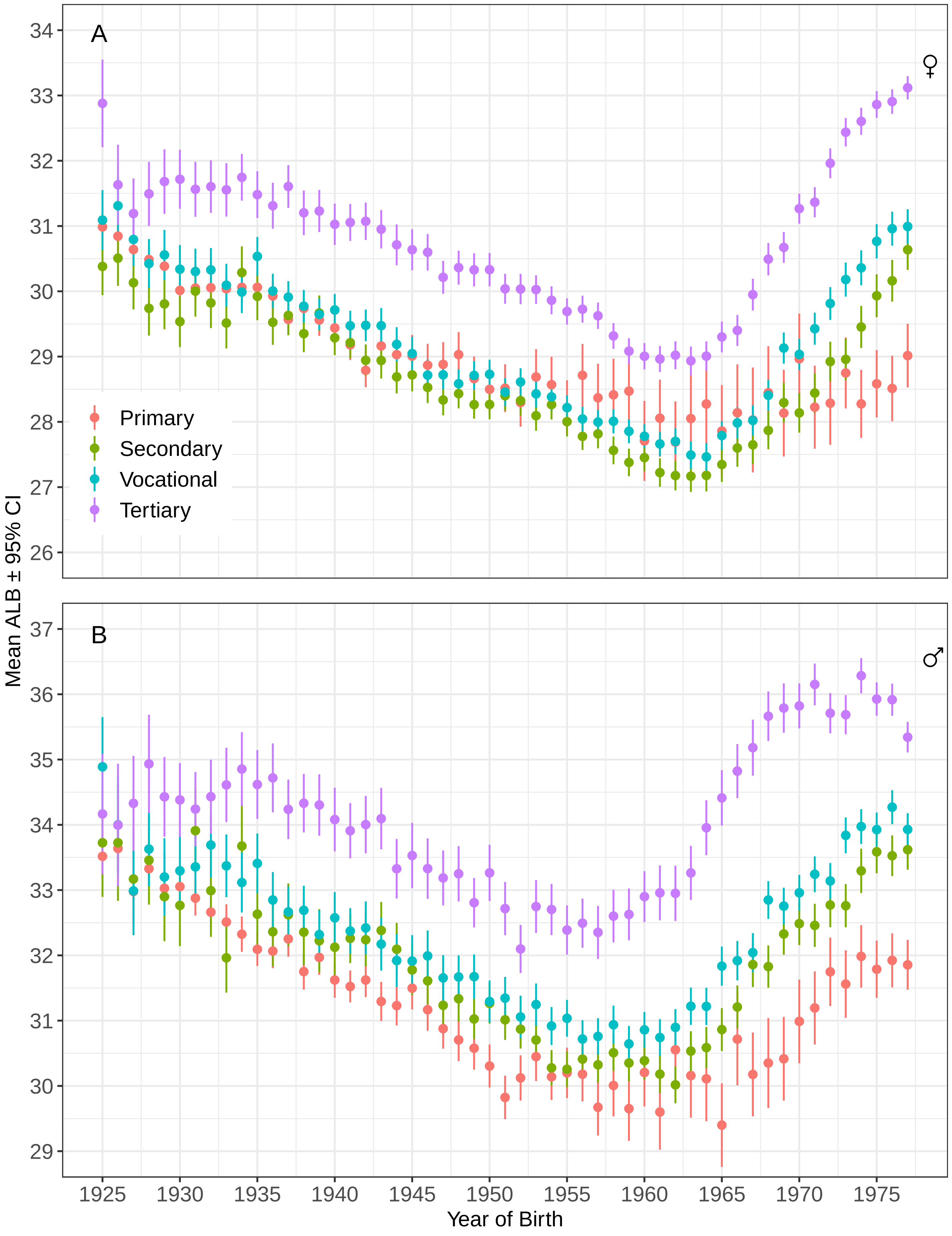

Figure S3. Birth cohort averages with 95% CI for age of last birth (ALB, years).

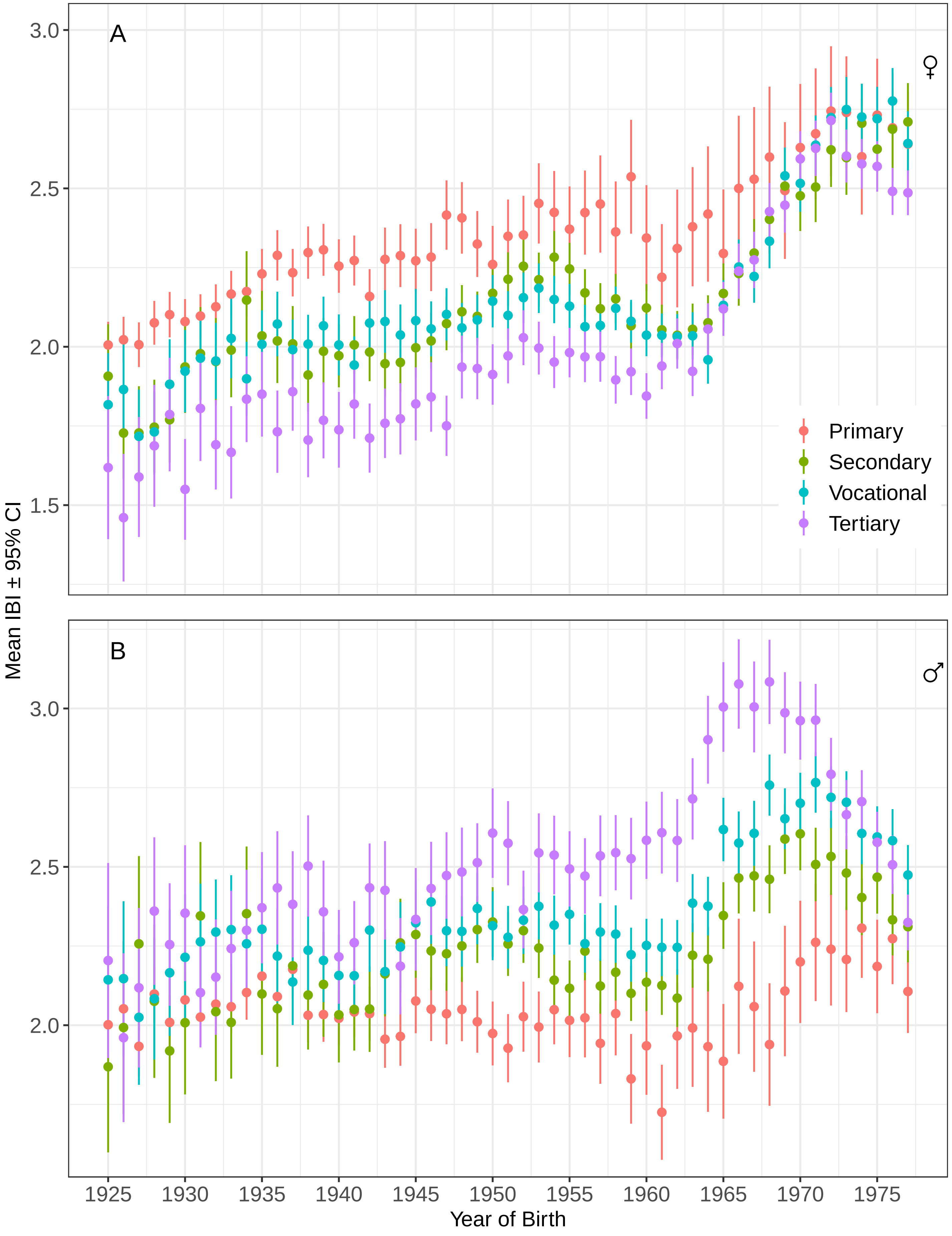

Figure S4. Birth cohort averages with 95% CI for average interbirth intervals (IBI, years).

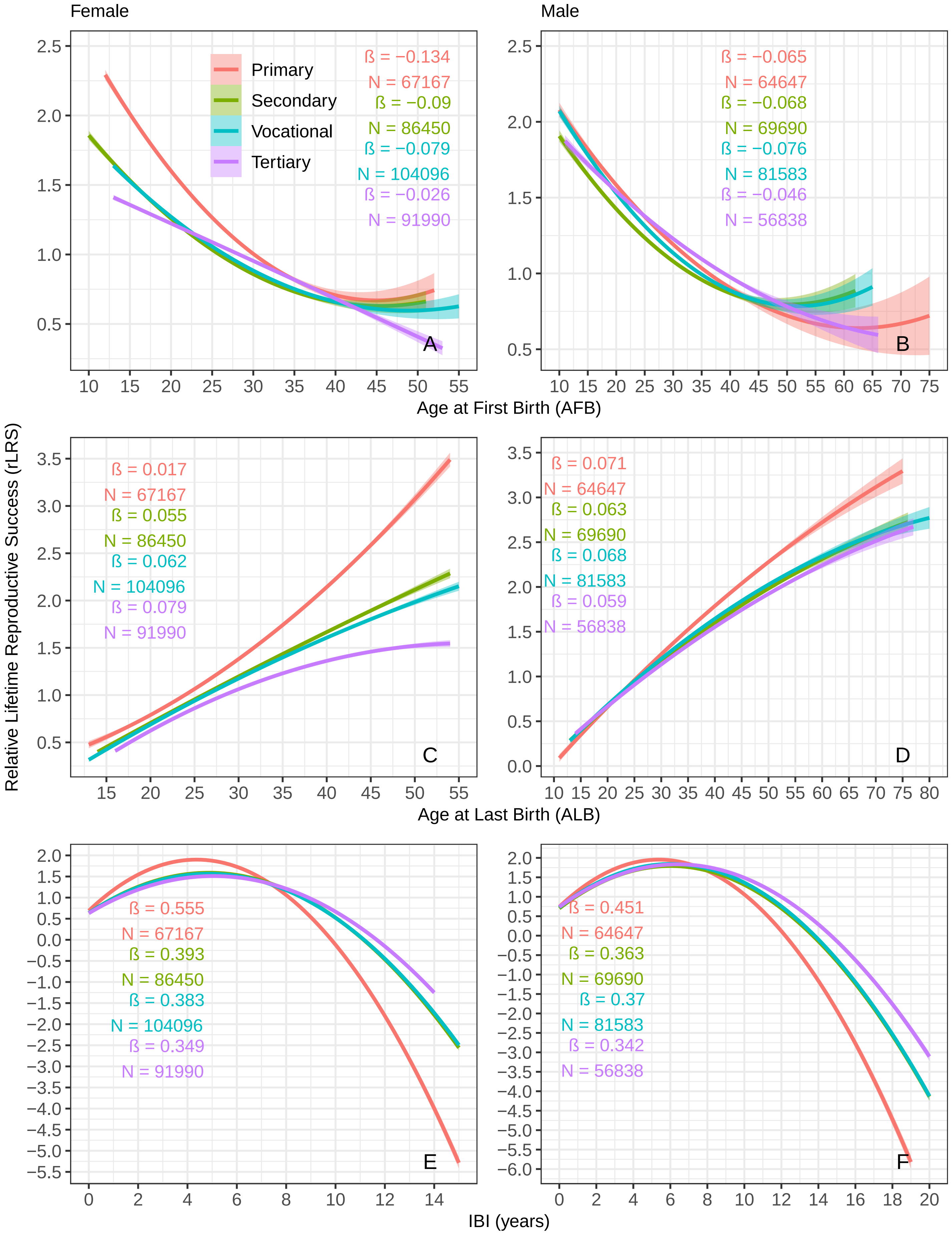

Figure S5. Quadradic selection curves with 95% CI for un-standardized values of reproductive timing traits over the whole study period. βs are linear selection gradients (β_Q) estimated from the quadratic regression model: rLRS = α + β_Q (trait zscore) + 1/2 γ(trait zscore)^2 + ε.

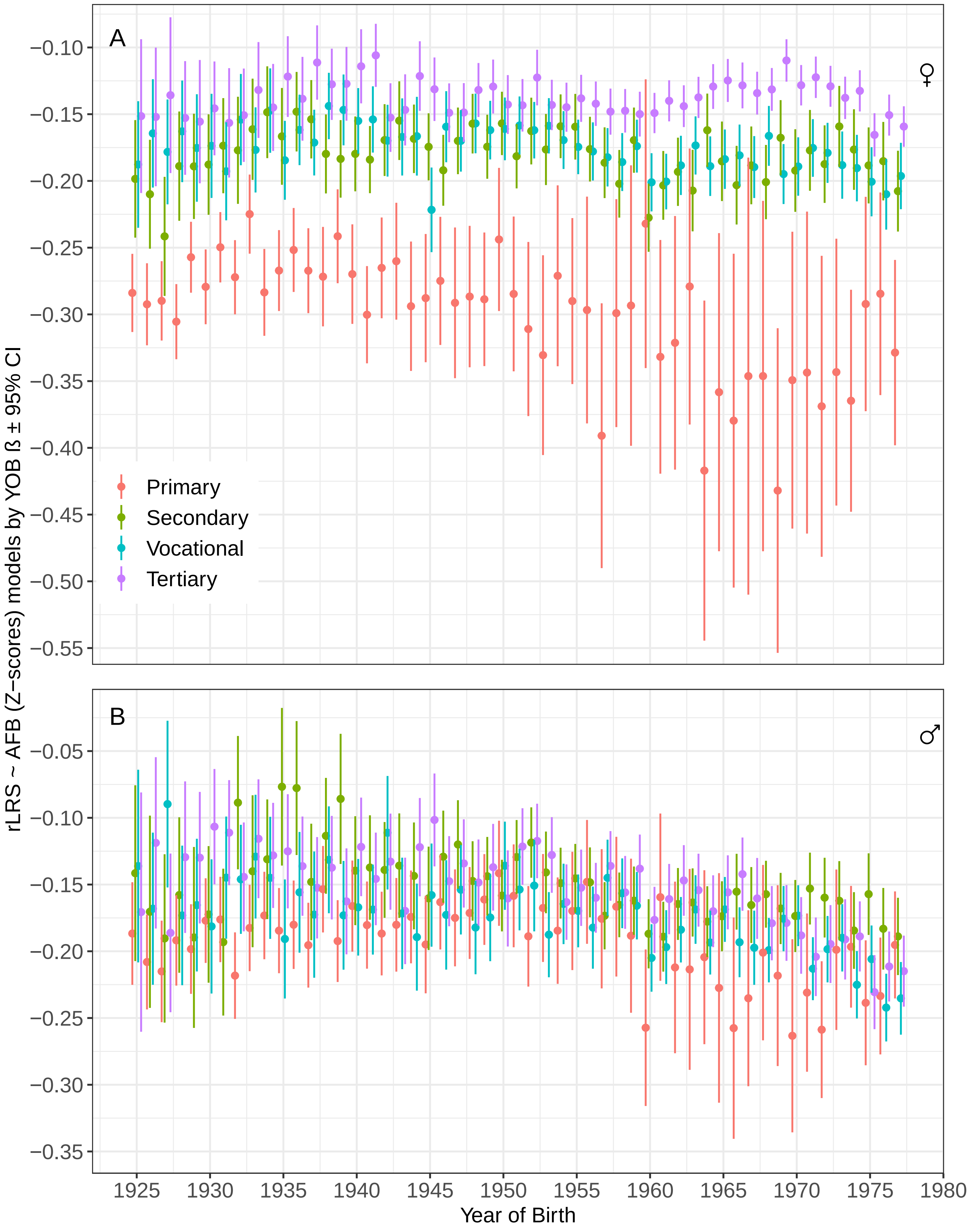

Figure S6. Cohort average linear selection gradients (β and 95% CI) on age of first birth by sex and educational attainment levels.

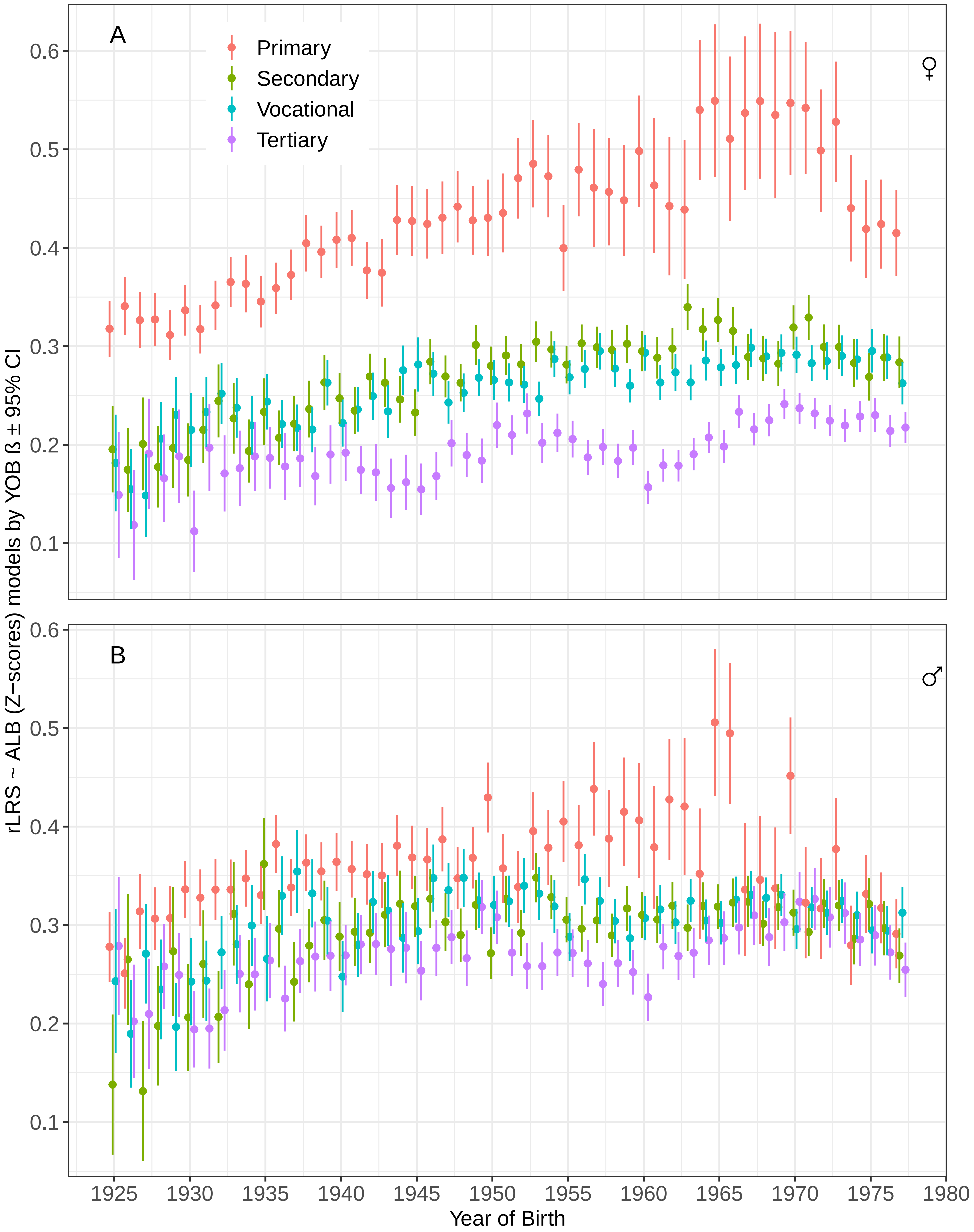

Figure S7. Cohort average linear selection gradients (β and 95% CI) on age of last birth by sex and educational attainment levels.

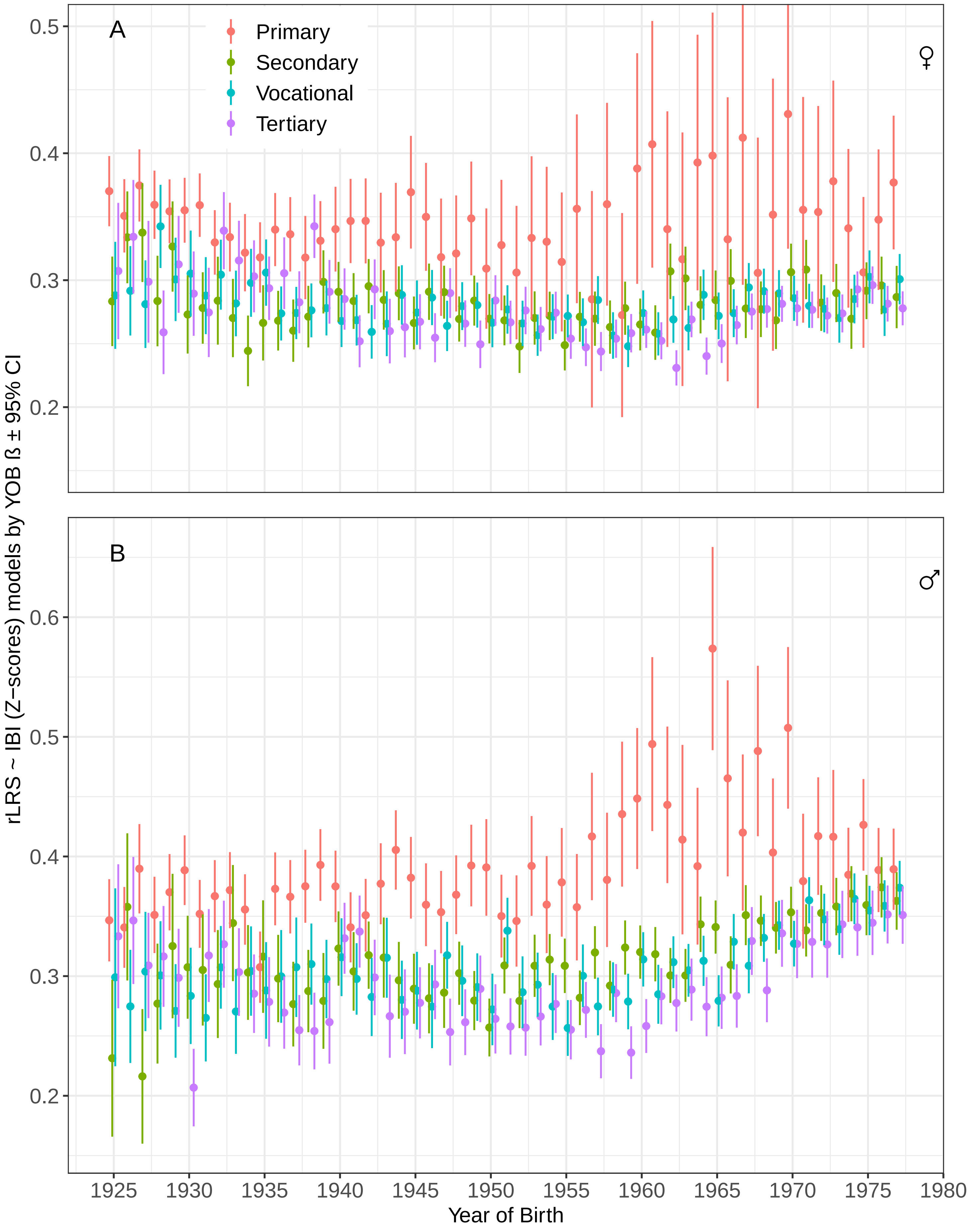

Figure S8. Cohort average linear selection gradients (β and 95% CI) on average interbirth intervals by sex and educational attainment levels.
